# *In vitro* studies implicate an imbalanced activation of dendritic cells in the pathogenesis of murine autoimmune pancreatitis

**DOI:** 10.18632/oncotarget.10265

**Published:** 2016-06-23

**Authors:** Luise Borufka, Erik Volmer, Sarah Müller, Robby Engelmann, Horst Nizze, Saleh Ibrahim, Robert Jaster

**Affiliations:** ^1^ Department of Medicine II, Division of Gastroenterology, Rostock University Medical Center, Rostock, Germany; ^2^ Institute of Immunology and Core Facility for Cell Sorting & Cell Analysis, Rostock University Medical Center, Rostock, Germany; ^3^ Institute of Pathology, Rostock University Medical Center, Rostock, Germany; ^4^ Institute of Experimental Dermatology, University of Lübeck, Lübeck, Germany

**Keywords:** autoimmune pancreatitis, mouse model, dendritic cells, cell culture, gene expression, Immunology and Microbiology Section, Immune response, Immunity

## Abstract

**Objectives:**

MRL/MpJ mice spontaneously develop an autoimmune pancreatitis (AIP) and are widely used as a model to study the genetic, molecular and immunological basis of the disease. Here, we have addressed the question whether distinctive features of their dendritic cells (DCs) may predispose MRL/MpJ mice to the chronic inflammation.

**Methods:**

Pancreatic lesions were analyzed employing histological methods. Cohorts of young (healthy) MRL/MpJ mice, adult (sick) individuals, and AIP-resistant CAST/EiJ mice were used to establish cultures of bone marrow (BM)-derived conventional DCs (cDCs). The cells were subsequently characterized regarding the expression profile of CD markers and selected genes, proliferative activity as well as cytokine secretion.

**Results:**

In pancreatic lesions, large numbers of cells expressing the murine DC marker CD11c were detected in close spatial proximity to CD3^+^ cells. A high percentage of BM-derived cDCs from adult MRL/MpJ mice expressed typical markers of DC maturation (such as CD83) already prior to a treatment with lipopolysaccharide (LPS). After LPS-stimulation, cDC cultures of both MRL/MpJ mouse cohorts contained more mature cells, proliferated at a higher rate and secreted less interleukin-10 (but also less pro-inflammatory cytokines) than cultures of CAST/EiJ mice. Compared with corresponding cultures of the control strain, LPS-free cultured cDCs from MRL/MpJ mice expressed less mRNA of the inhibitory receptor *triggering receptor expressed on myeloid cells 2* (trem2).

**Conclusions:**

BM-derived cDCs from AIP-prone MRL/MpJ mice display functional features that are compatible with the hypothesis of an imbalanced DC activation in the context of murine AIP.

## INTRODUCTION

Autoimmune pancreatitis (AIP) represents a rare form of chronic pancreatitis that has nevertheless gained significant clinical attention in recent years since it is frequently misdiagnosed as pancreatic cancer but can be efficiently treated with steroids upon correct diagnosis [1, 2]. There are at least two different subtypes of AIP, type 1 and type 2, which can be distinguished based on their histopathological patterns: AIP type 1 is characterized by a dense infiltration of the pancreatic tissue with lymphocytes and IgG4^+^ plasma cells and is considered as the pancreatic manifestation of IgG4-related disease. In contrast, the pathognomonic findings in AIP type 2 are granulocytic epithelial lesions. Independent of the subtype, progression of AIP is frequently associated with formation of inflammatory pseudotumors and a replacement of pancreatic tissue by connective tissue (fibrosis) as a result of inadequate wound healing [2, 3].

Like other autoimmune diseases, AIP is considered as a multifactorial disease that is caused by the interplay of predisposing genetic factors and environmental effects [1]. Genetic risk factors for the development or recurrence of AIP include the HLA serotypes DRB1*0405 and DQB1*0401 (in a Japanese population) [4], a mutation of *DQbeta1* found in patients from Korea [5], and single nucleotide polymorphisms in several non-HLA genes [6–10]. Using a mouse model of spontaneous AIP, MRL/MpJ [11], we recently mapped 6 quantitative trait loci (QTLs), termed AIP1-AIP6, that contain further putative candidate genes [12].

The immunological triggers of AIP are largely unknown yet. It has been proposed that the production of antibodies against the plasminogen binding protein of *H. pylori* may lead to an autoimmune response against pancreatic acinar cells *via* molecular mimicry [13, 14], but this hypothesis remains to be validated. The pathogenetic role of IgG4 (AIP type 1) and the various autoantibodies (both subtypes) is still uncertain, but a crucial involvement of B-cells/plasma cells has nevertheless been convincingly demonstrated through the clear therapeutic efficiency of a B-cell depletion with anti-CD20 antibodies [15].

In addition to B-cells, immune responses of several subtypes of T-cells, including both T-helper (Th) 1 and Th2 cells, have been implicated in the progression of AIP [1, 16–18]. Furthermore, increased numbers of regulatory T-cells have been detected in peripheral blood and pancreatic tissue of AIP patients [19, 20], and own studies in the MRL/MpJ mouse model have provided experimental evidence for a regulatory function of this cell type as well as a key role of effector T-cells in the development of murine AIP [20, 21]. Most recently, we have identified in the same mouse strain CD4^+^/CD44^high^ memory T-cells as an important link between genetic susceptibility and emergence of the disease [22]. Noteworthy, pancreatic autoimmune lesions have been shown in some mouse models to progress with increasing age [23], a phenomenon that might, at least in part, be related to a less efficient action of inhibitory immune cells in aged animals.

Dendritic cells (DCs) are powerful antigen-presenting cells which are involved in the initiation and regulation of both innate and adaptive immune responses. On the other hand, a *dysregulated* DC activation has been implicated in the induction of a broad range of autoimmune manifestations; e.g., through an inappropriate activation and effector differentiation of relevant T-cell populations [24].

DCs comprise two major classes, conventional DCs (cDCs) and plasmacytoid DCs (pDCs). In the only study that has addressed the specific role of pDCs in the context of AIP to date, Arai *et al.* could recently show that pDC activation and the subsequent production of interferon (IFN)-α are prominent features of both murine AIP and human IgG4-related pancreatitis [25], as they are also in a number of other human autoimmune diseases [24]. Importantly, pDCs were not only present in the inflamed pancreatic tissue, but were also found indispensable for the generation of IgG4 responses in patients with IgG4-related AIP [25].

Here, we again took advantage of the MRL/MpJ mouse model to study another potential implication of DCs in the pathogenesis of AIP: the possibility that specific functional features and defects of DCs may favor the development of the disease. The investigations were encouraged by the results of our genetic studies mentioned above [12], which had located a putative candidate gene within AIP5, *C-type lectin domain family 4, member a2* (*clec4a2*), that is known as a negative regulator of DC expansion and crucially involved in maintaining the homeostasis of the immune system [26]. Another gene with regulatory function in DCs, *cytohesin-interacting protein* (*cytip*), lies within the QTL AIP1 on chromosome 2. *Cytip* has been shown to be essential for the dissolving of DC-T-cell conjugates formed during the priming phase of an immune response [27]. Finally, a third gene with a regulatory action in DCs, *triggering receptor expressed on myeloid cells* (*trem2*), was identified within a region on chromosome 17 that contains another putative QTL (S.I. and R.J., unpublished data). *Trem2*, which is mainly expressed on myeloid cells (including myeloid DCs) has emerged as an important anti-inflammatory receptor and previously been implicated in the inhibition of autoimmune diseases such as experimental autoimmune encephalomyelitis [28, 29].

Using bone marrow (BM) as a source of cDCs, we found differences in maturation, proliferation, cytokine secretion and gene expression between AIP-prone MRL/MpJ mice and an AIP-resistant control strain, CAST/EiJ, that are compatible with the hypothesis of a dysregulated DC activation in the context of murine AIP.

## RESULTS

### Histological evidence for a role of DCs in the pathogenesis of murine AIP

**Figure 1:**
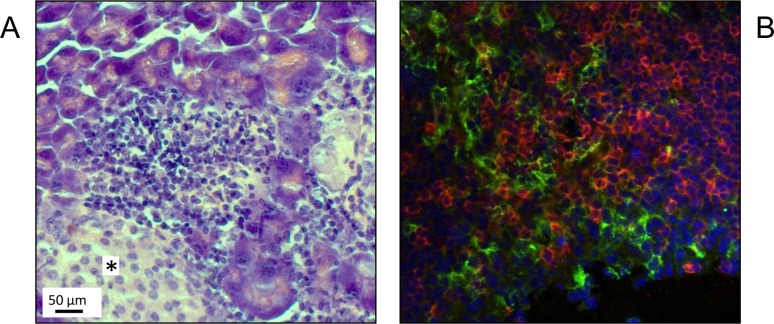
Presence of CD11c^**+**^ cells in pancreatic lesions of MRL/MpJ mice Pancreatic tissue from an adult MRL/MpJ mouse was subjected to histological and immunofluorescence studies. **A.** H&E staining shows pathological changes typical of AIP, stage 2 (infiltrates of mononuclear cells; beginning destruction of exocrine tissue). Note that the pancreatic islet, indicated by an asterisk, is free of infiltrate. **B.** Immunofluorescence staining of CD3^+^ lymphocytes (red) and CD11c^+^ cells (green) within an inflammatory pancreatic lesion revealed a close spatial proximity of both cell types. Counterstaining was performed employing DAPI (blue) (original magnification 60x).

MRL/MpJ mice, especially female individuals, spontaneously develop AIP at an age of at least 6 months [11, 12]. Histopathologically, the disease is characterized by focal (later on, diffuse) interstitial infiltrates of mononuclear cells and a progressive destruction of the exocrine pancreatic tissue (Figure 1A). In agreement with our previous studies [12, 20, 21, 30], infiltrates of inflammatory cells were found to consist predominantly of T-lymphocytes (Figure 1B). Interestingly, in close spatial proximity to cells expressing the T-cell receptor CD3, high numbers of cells that stained positive for the murine DC marker CD11c were detected (Figure 1B). Together with the results of our previous genetic studies [12], these observations prompted us to ask if functional peculiarities of their DCs may contribute to the high susceptibility of MRL/MpJ mice to AIP.

### BM-derived cDCs of diseased MRL/MpJ mice exhibit a mature phenotype

To obtain sufficient cell numbers for functional studies, cDCs were generated *in vitro* from BM cells employing standard methods [31]. After 9 days of incubation with granulocyte-macrophage colony-stimulating factor (GM-CSF), cultures of cDCs were received that contained, on average, roughly 90 % CD11c^+^ cells (range for the different experimental groups: 81.7-96.8 %). cDC cultures were established from male and female individuals of the following three mouse cohorts:

(1) 12-15 weeks-old, AIP-prone but still healthy MRL/MpJ mice (subsequently termed young MRL/MpJ mice; AIP score of 0 in all animals)
(2) 34-51 weeks-old MRL/MpJ mice with progressive AIP (subsequently termed adult MRL/MpJ mice; averaged AIP score for both genders together: 1.9)
(3) 38-45 weeks-old mice of the AIP-resistant control strain CAST/EiJ (AIP score of 0 in all animals)

Further details of the groups are given in Table 1. Noteworthy, in this experimental series AIP was only slightly more severe in adult female MRL/MpJ mice than in male individuals; an observation that differs gradually from previous findings by us and others [11, 12] and will be considered in follow-up investigations. In our studies with cDCs, sex-dependent differences were not detected at all (data not shown). In all subsequently described figures, data of male and female mice are therefore presented in a combined manner.

**Figure 2:**
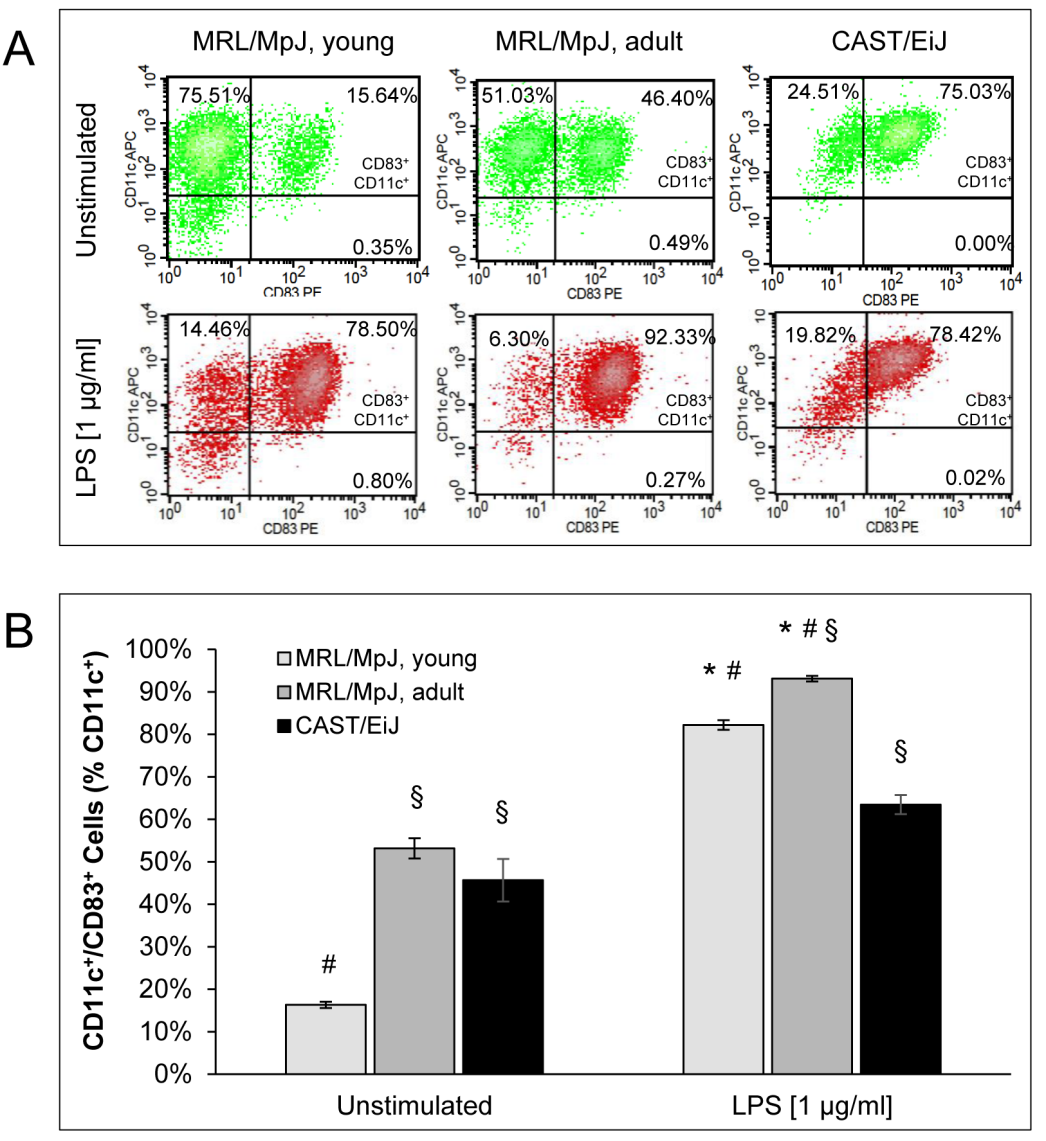
Coexpression of CD11c and CD83 by BM-derived cDCs BM cells of the indicated mouse cohorts were cultured for 9 days with GM-CSF (20 ng/ml) to generate BM-derived cDCs. Afterwards, the cells were stimulated for 24 h with LPS (1 μg/ml) as indicated. Expression of the cell surface proteins CD11c and CD83 was studied by flow cytometry. **A.** Representative dot plots of flow cytometric analyses are shown for all experimental groups and culture conditions (with and without LPS, respectively). **B.** CD11c/CD83 double-positive cells are indicated as percentage of all CD11c^+^ cells (= 100 %). Data are presented as mean ± SEM (*n* ≥ 12 cultures from different mice); *P < 0.05 *versus* cDCs of the same experimental group cultured without LPS; ^§^*P* < 0.05 *versus* identically cultured cDCs of young MRL/MpJ mice; ^#^*P* < 0.05 *versus* identically cultured cDCs of CAST/EiJ mice.

CD11c^+^ cells of all mouse cohorts expressed the DC-typical surface markers MHC-II, CD40, CD80, CD83 and CD86. Strikingly, the percentages of CD11c^+^/CD83^+^ (Figure 2), CD11c^+^/CD40^high^, CD11c^+^/CD80^high^ and CD11c^+^/CD86^high^ cells (all Figure S1) were significantly higher in cDC cultures of adult (sick) MRL/MpJ mice than in those from young (healthy) animals of the same strain. cDC cultures of AIP-resistant CAST/EiJ mice displayed intermediate values for all four surface marker combinations mentioned above.

In both cDC cultures from young and adult MRL/MpJ mice, stimulation with the trigger lipopolysaccharide (LPS) strongly increased the percentage of CD11c^+^ cells which expressed the maturation marker CD83 (Figure 2). At the same time, increased percentages of CD11c^+^/CD40^high^, CD11c^+^/CD80^high^ and CD11c^+^/CD86^high^ cells (all Figure S1) were detected. Although the differences between cDCs of young and adult MRL/MpJ mice declined upon LPS treatment, they still remained statistically significant. Intriguingly, LPS-stimulated cDCs of CAST/EiJ mice showed the lowest values for all four marker combinations that were investigated.

**Table 1:** Characteristics of the mouse cohorts

Mouse Strain	Cohort	Sex	Number of Mice	Age (Weeks ± SEM)	AIP (Score ± SEM)
MRL/MpJ, young	1	male	7	12.9 ± 0.2	0.0 ± 0.0
MRL/MpJ, young	1	female	7	13.7 ± 0.4	0.0 ± 0.0
MRL/MpJ, young	1	male/female	14	13.3 ± 0.2	0.0 ± 0.0
MRL/MpJ, adult	2	male	10	35.3 ± 0.3	1.7 ± 0.3
MRL/MpJ, adult	2	female	13	38.8 ± 1.8	2.1 ± 0.2
MRL/MpJ, adult	2	male/female	23	37.3 ± 1.1	1.9 ± 0.2
CAST/EiJ	3	male	5	42.4 ± 1.1	0.0 ± 0.0
CAST/EiJ	3	female	11	41.7 ± 0.8	0.0 ± 0.0
CAST/EiJ	3	male/female	16	41.9 ± 0.6	0.0 ± 0.0

Mice numbers refer to the total number of animals that were included into the study. The number of mice in the individual experiments is indicated in the respective figure legends.

**Figure 3:**
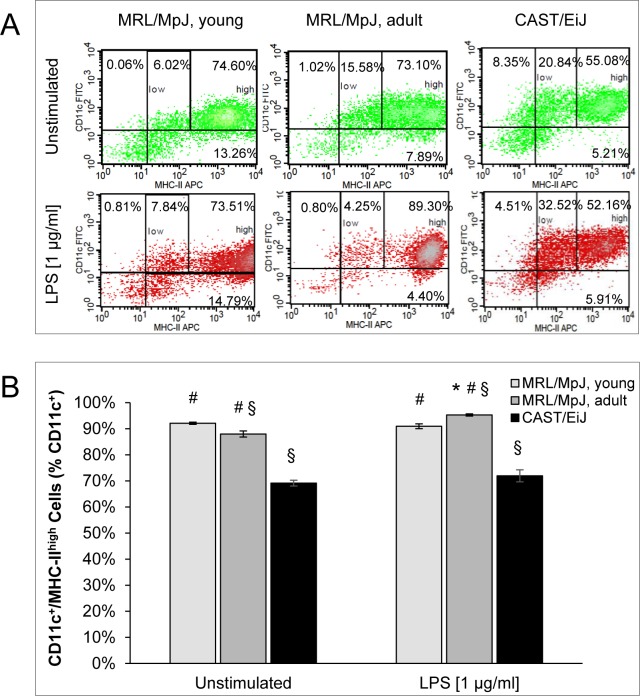
Coexpression of CD11c and MHC-II by BM-derived cDCs For experimental details, please refer to Figure 2. **A.** Representative dot plots of flow cytometric analyses are shown for all experimental groups and culture conditions (with and without LPS, respectively). **B.** CD11c^+^/MHC-II^high^ cells are indicated as percentage of all CD11c^+^ cells (= 100 %). Data are presented as mean ± SEM (*n* ≥ 12 cultures from different mice); *P < 0.05 *versus* cDCs of the same experimental group cultured without LPS; ^§^*P* < 0.05 *versus* identically cultured cDCs of young MRL/MpJ mice; ^#^*P* < 0.05 *versus* identically cultured cDCs of CAST/EiJ mice.

The expression profile of MHC-II differed from the ones of the other markers in some regards (Figure 3). Specifically, there were only small quantitative differences between cDCs of young and adult MRL/MpJ mice. Somewhat unexpectedly, LPS treatment increased the percentage of MHC-II^high^ cells in cDC cultures of adult MRL/MpJ mice only. cDCs of CAST/EiJ mice displayed the lowest levels of MHC-II expression both in the presence and absence of LPS.

Together, these data suggest that many BM-derived cDCs of adult MRL/MpJ mice exhibit a mature phenotype even prior to stimulation with LPS. Furthermore, cDCs of MRL/MpJ mice of both age groups are highly sensitive to a LPS-dependent induction of maturation.

### Growth characteristics and cytokine expression profiles of BM-derived cDC cultures

**Figure 4:**
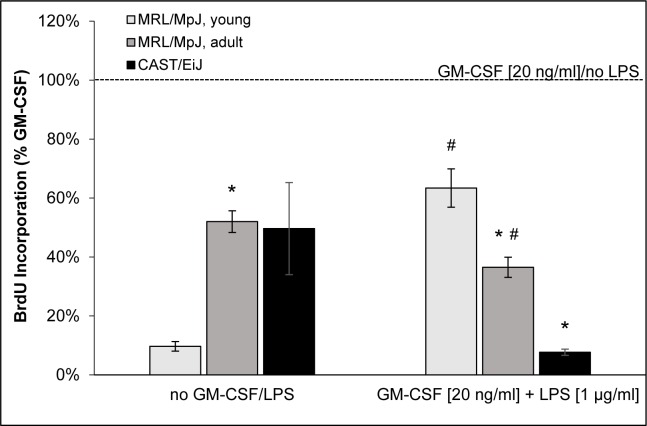
BrdU incorporation of BM-derived cDCs On day 9 of routine culture, cDCs of young MRL/MpJ mice, adult individuals of the same strain and CAST/EiJ mice were cultured for 24 h with GM-CSF and LPS as indicated. Pretreated cells were labeled with BrdU for another 24 hours and proliferation was assessed with the BrdU DNA incorporation assay. One hundred percent BrdU incorporation corresponds to cDCs of the respective strain that were cultured under standard culture conditions (GM-CSF at 20 ng/ml, no LPS). Data are presented as mean ± SEM (*n* ≥ 11 cultures from different mice); *P < 0.05 *versus* identically cultured cDCs of young MRL/MpJ mice; ^#^*P* < 0.05 *versus* identically cultured cDCs of CAST/EiJ mice.

5-bromo-2′-deoxy-uridine (BrdU) incorporation assays indicated that cDC cultures of all mouse cohorts synthesized DNA in a GM-CSF-dependent manner (Figure 4). Both GM-CSF-free culture conditions and the induction of maturation by LPS treatment were associated with a reduction of cell proliferation. Interestingly, however, cDCs of adult MRL/MpJ mice were much less sensitive to the withdrawal of GM-CSF than cells of young mice. Furthermore, cDCs from MRL/MpJ mice of both age groups were more robust against the inhibitory effect of LPS than cells of the AIP-resistant strain CAST/EiJ (with cDCs of young MRL/MpJ showing the highest rate of DNA synthesis under these conditions).

**Figure 5:**
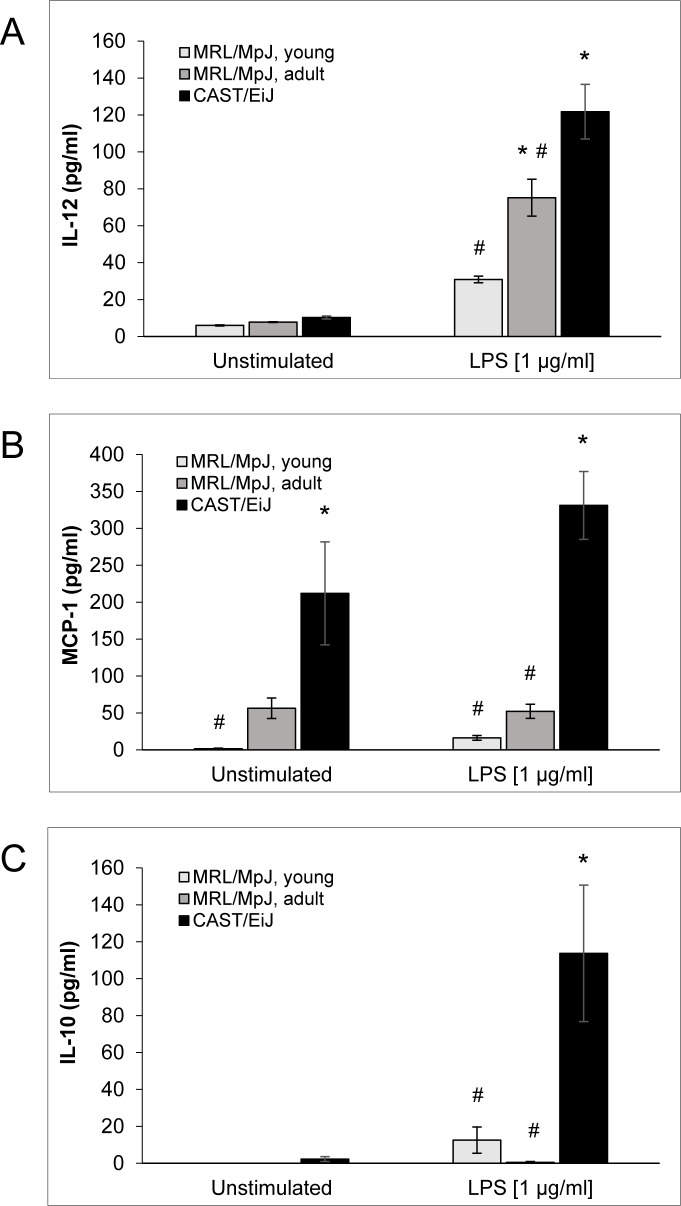
Cytokine levels in cDC culture supernatants The supernatants of BM-derived cDC cultures from the indicated experimental groups were collected on day 10 after routine culture with GM-CSF alone (*unstimulated*; left columns) and after 24 h of stimulation with LPS (1 μg/ml; right columns). The levels of **A.** IL-12 were determined by ELISA, whereas concentrations of **B.** MCP-1 and **C.** IL-10 were measured using the LEGENDplex Multi-Analyte Flow Assay Kit. Data are presented as mean ± SEM (*n* = 6 cultures from different mice for MCP-1 and IL-10; *n* ≥ 14 for IL-12); *P < 0.05 *versus* identically cultured cDCs of young MRL/MpJ mice; ^#^*P* < 0.05 *versus* identically cultured cDCs of CAST/EiJ mice. There were no significant differences between young and adult MRL/MpJ mice for any cytokine and experimental condition.

To further assess the activation status of the different cDC populations, the concentrations of various DC-typical cytokines in culture supernatants were determined (Figure 5 and Figure S2). Consistent with the maturation-inducing effect of LPS, there was a general trend towards higher cytokine levels in LPS-stimulated cultures of all experimental groups. Unexpectedly, it was also observed that LPS-treated cDCs of AIP-resistant CAST/EiJ mice secreted more interleukin (IL)-12, a key factor in T-cell activation and their differentiation into Th1-cells [32], than cDCs from MRL/MpJ mice of both age groups (Figure 5A). Similar observations were made for monocyte chemotactic protein (MCP)-1 (Figure 5B) and proinflammatory cytokines (IL-1β, IL-6 and TNF-α; Figure S2). Most interestingly, however, cDCs of CAST/EiJ mice also secreted the by far highest amounts of anti-inflammatory IL-10 (Figure 5C).

A comparison of LPS-free cultured cDCs from young (healthy) and adult (sick) MRL/MpJ mice revealed no significant differences, but a consistent trend towards higher levels of the proinflammatory mediators (MCP-1, IL-1α, IL-6 and TNF-α) in the latter group (Figure 5 and Figure S2). In the presence of LPS, cDCs of adult MRL/MpJ mice secreted significantly more IL-12 than cells of young mice (Figure 5A), whereas the levels of the other cytokines neither showed significant differences, nor a consistent trend.

### Gene expression profiles of BM-derived cDCs from MRL/MpJ and CAST/EiJ mice

In this part of the study, we focused on the three putative candidate genes for the genetic control of murine AIP mentioned above, *clec4a2*, *trem2* and *cytip*, which all have previously been implicated in the regulation of DC function.

**Figure 6:**
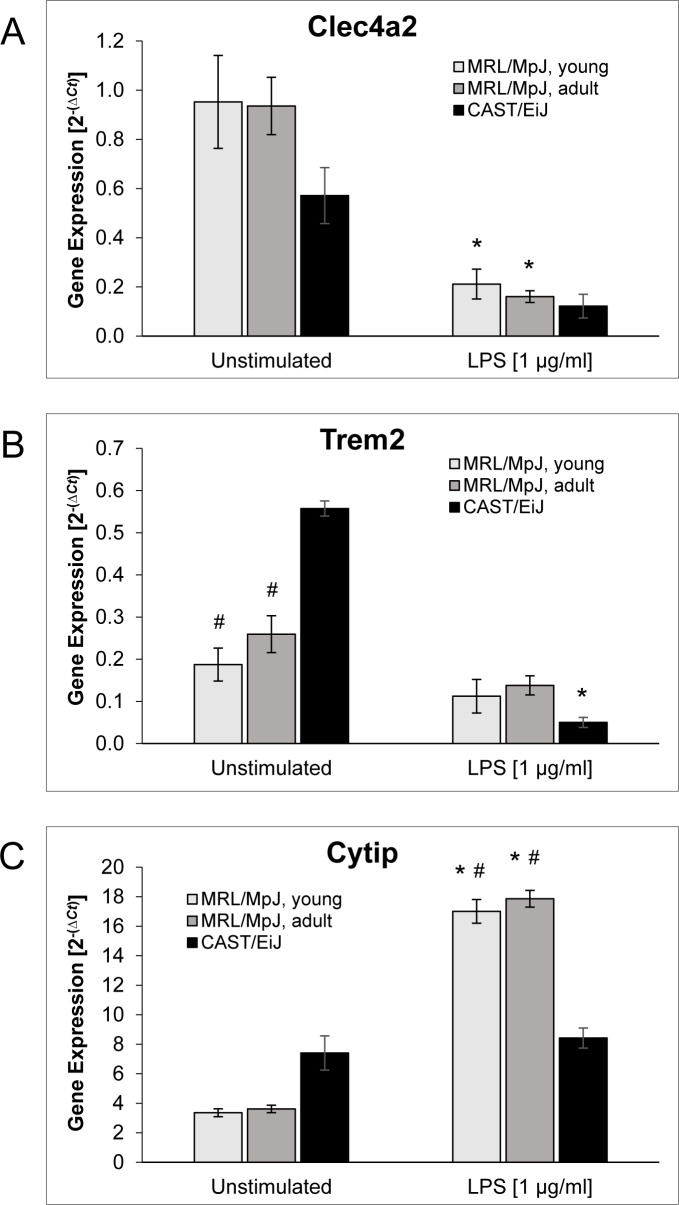
Gene expression of BM-derived cDCs BM cells of the indicated mouse cohorts were cultured for 9 days with GM-CSF (20 ng/ml) to generate BM-derived cDCs. Afterwards, the cells were exposed to LPS (1 μg/ml; right columns) or left unstimulated (left columns). The mRNA expression of **A.** *clec4a2*, **B.** *trem2*, **C.** *cytip* and the housekeeping gene *hprt* was analyzed by real-time PCR, and relative amounts of target mRNA were calculated as described in the *Methods* section. Data of *n* ≥ 6 independent cultures from different mice were used to calculate mean ± SEM. *P < 0.05 *versus* cDCs of the same experimental group cultured without LPS; ^#^*P* < 0.05 *versus* identically cultured cDCs of CAST/EiJ mice. There were no significant differences between the mRNA levels in cDCs of young and adult MRL/MpJ mice for any gene and treatment protocol.

Expression of *clec4a2* (Figure 6A) did not differ significantly between cDCs of the three mouse cohorts, independent of the presence or absence of LPS. In response to LPS stimulation, *clec4a2* mRNA levels dropped significantly in cDCs from young and adult MRL/MpJ mice, but also by trend in cDCs from CAST/EiJ mice.

LPS-free cultured cDCs from both cohorts of MRL/MpJ mice expressed significantly less *trem2* mRNA than cDCs of CAST/EiJ mice (Figure 6B). Induction of maturation by LPS stimulation was associated with a significant decrease of *trem2* mRNA levels in cDCs of the AIP-resistant strain only.

With respect to the expression of *cytip* (Figure 6C), unstimulated cDCs of the three mouse cohorts displayed no significant differences. Upon challenge with LPS, however, cDCs of both young and adult MRL/MpJ mice expressed significantly more *cytip* mRNA than the control strain.

### Effects of cDCs on syngeneic T-cells *in vitro*

**Figure 7:**
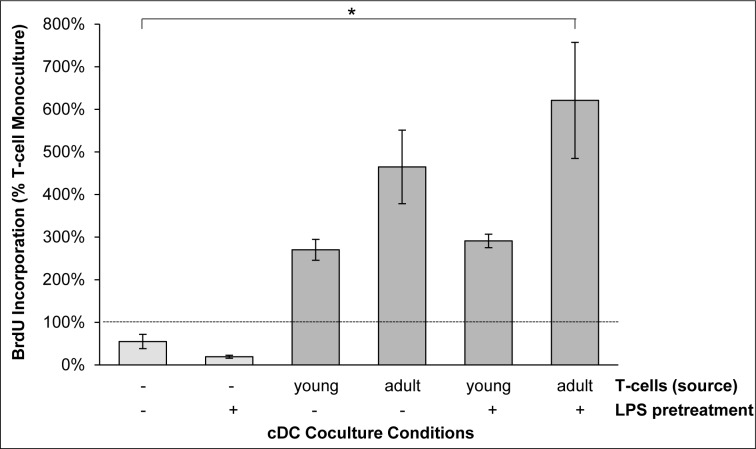
BrdU incorporation of cDC/T-cell cocultures Irradiated cDCs of young MRL/MpJ mice (pretreated with LPS as indicated) were cocultured with splenic T-cells from young and adult MRL/MpJ mice for 48 h as indicated. Afterwards, the cells were labeled with BrdU for another 24 h and proliferation was assessed with the BrdU DNA incorporation assay. The corresponding monocultures of T-cells from young and adult MRL/MpJ mice were set as one hundred percent. For monocultures of DCs (columns 1 and 2), all T-cell monocultures together were used as reference (100%). Data are presented as mean ± SEM (*n* ≥ 6 separate cultures); *P < 0.05 *versus* corresponding monocultures of T-cells.

To gain additional insights into cDC functions in the context of murine AIP, coculture experiments were performed (Figure 7). Therefore, irradiated cDCs from MRL/MpJ mice were employed that did not proliferate and incorporated BrdU at a low rate only (left two columns). Both LPS-pretreated and untreated cDCs efficiently triggered DNA-synthesis of syngeneic T-cells from young and adult mice (right four columns); a finding that is in agreement with previous studies and not specific for the AIP-prone MRL/MpJ strain [33]. We noticed that there was a trend towards a stronger proliferation of T-cells from diseased (adult) mice compared with healthy (young) individuals. The trend was observed both for LPS-treated and untreated DCs, but the level of statistical significance was missed.

## DISCUSSION

DCs are crucially involved in the pathogenesis of various human and experimental autoimmune diseases, including type 1 diabetes mellitus, cardiac and neurological autoimmune disorders, systemic lupus erythematosus (SLE), and psoriasis (reviewed in [24]). With respect to AIP, however, only few clinical and experimental data on the pathogenetic role of DCs are available yet. In our studies with AIP-susceptible MRL/MpJ mice, we found, in agreement with Arai *et al.* [25], DCs in large numbers within pancreatic lesions of diseased animals. The cells were located in immediate vicinity to CD3^+^ cells, suggesting that they might be involved in the priming of effector T-cells. In most cases that have been studied, initiation of autoimmunity, however, seems to depend not on the mere gain or loss of DCs, but on changes in DC functionality [24]. To gain mechanistic insights into DC functions in our model, we therefore performed *in vitro* studies with BM-derived cDCs from AIP-prone but still healthy MRL/MpJ mice, diseased individuals as well as an AIP-resistant strain, CAST/EiJ. The perhaps most intriguing finding was that many cDCs from MRL/MpJ mice with an advanced AIP displayed a mature phenotype (CD11c^+^/CD83^+^/MHC-II^high^/CD40^high^/CD80^high^/CD86^high^) already prior to incubation with LPS. Furthermore, LPS-treated cDC cultures of both MRL/MpJ mouse cohorts contained more mature cells and proliferated at a higher rate than cDC cultures of the control strain.

Spontaneous maturation of DCs is a phenomenon that has previously been observed in other models of autoimmunity and linked to the pathogenesis of the diseases [24, 34–37]. Thus, in mice with a targeted deletion of either *A20* (*Tnfaip3*) or *SHP1* (*Ptpn6*), two cell-intrinsic negative regulators of DC function, DC maturation triggers a polyclonal immune activation [34–37]. Subsequently, both mouse models develop a SLE-like disease. In addition, *A20* knockout mice display symptoms of lymphocyte-dependent colitis, seronegative ankylosing arthritis and enthesitis; conditions, which are stereotypical of human inflammatory bowel disease [35]. With respect to AIP, it has been shown in a genetic mouse model that loss of TGFβ signalling in S100A4-positive DCs is an important factor in the development of the disease [38].

DCs from *A20*- and *SHP1*-deficient mice were also reported to produce increased amounts of pro-inflammatory cytokines [35–37]. Unexpectedly, cDCs from both healthy and sick MRL/MpJ mice secreted *less* MCP-1, IL-1α, TNF-α and IL-6 (as well as less IL-12) than cells of AIP-resistant CAST/EiJ mice. Noteworthy, however, culture supernatants of the AIP-susceptible mouse cohorts also contained lower concentrations of the pivotal anti-inflammatory cytokine IL-10. In conclusion, we suggest that spontaneous maturation of DCs, as well as an enhanced maturation in response to triggers, may play a pathogenetic role in the development of murine AIP. The possibility of an imbalanced production of pro- and anti-inflammatory cytokines needs to be studied further, since our results are, in this regard, heterogeneous.

In our studies, we have employed *in vitro* generated cDCs (rather than DCs from lymphatic or pancreatic tissue, which are available in small numbers only). The fact that these BM-derived cDCs from AIP-prone mice and controls nevertheless display distinct functional features, points to genetically fixed differences of DC functionality between the strains. Support for this hypothesis comes from our genetic studies, where genes with regulatory functions in DCs were among the putative candidate genes ([12] and unpublished data). In this investigation, we focused on *clec4a2*, *trem2* and *cytip* by analyzing the expression of these genes at the level of mRNA. LPS-free cultured cDCs from both young (still healthy) and adult (sick) MRL/MpJ mice were found to express the inhibitory receptor *trem2* [29] at a significantly lower level than corresponding cultures of the control strain. With respect to *cytip*, the increased expression of the gene in LPS-treated cDC cultures of both MRL/MpJ mouse cohorts deserves further attention, since the gene product has been implicated in the initiation of T-cell-mediated immune responses. In case of *clec4a2*, no significant differences between the mouse cohorts were observed.

It thas been shown that murine DCs are capable of activating syngeneic naïve T-cells *in vitro* even in the absence of foreign antigen [33]. Indeed, we observed a cDC-dependent proliferation of syngeneic T-cells under coculture conditions. The subsequent experiments were based on the hypothesis that spleens of sick mice would contain more primed T-cells than spleens of still healthy individuals, and that this might influence T-cell proliferation upon contact with DCs. The results revealed no statistically significant differences between the experimental groups, but showed a trend and, therefore, encourage follow-up studies.

AIP represents a heterogeneous and multifactorial disease, which can only to some degree be reproduced in an animal model. MRL/MpJ mice, nevertheless, have proven a very useful tool to study various aspects of the autoimmune disorder. The disease develops spontaneously and with a high incidence in adult individuals (especially females) [11, 30], and can be further enhanced by triggers such as polyinosinic:polycytidylic acid (poly I:C) [39] and IFN-γ [20]. Pancreatic histopathology mimics human AIP type 1 [21], with the limitation that IgG4 is missing in mice. MRL/MpJ mice have not only been successfully used to study genetics and pathogenesis of AIP, but also to evaluate novel therapeutic approaches [21]. Nonetheless, the relevance of any result for the human situation needs to be addressed. This also applies to the key finding of this study: the observation that BM-derived cDCs from AIP-prone MRL/MpJ mice exhibit phenotypic characteristics that are compatible with the hypothesis of an imbalanced DC activation in the context of experimental AIP type 1.

## MATERIALS AND METHODS

### Mouse strains

MRL/MpJ and CAST/EiJ mice were purchased from Charles River Laboratories (Sulzfeld, Germany). MRL/MpJ mice spontaneously develop AIP, as previously described [11, 30] and further outlined in the results section, in an age and gender-specific manner. In contrast to previous studies by us and others [21, 25, 39], mice were *not* injected with the non-specific enhancer poly I:C to keep the autoimmune response as unbiased as possible.

As described before [12], CAST/EiJ mice were used as an AIP-resistant control strain. The animals were kept under specific pathogen-free conditions at a 12 h light/dark cycle with food and water ad libitum. All procedures were performed with adherence to the EU Directive 2010/63/EU for animal experiments and approved by the local governmental administrations (Landesamt für Landwirtschaft, Lebensmittelsicherheit und Fischerei Mecklenburg-Vorpommern).

### Histology and immunofluorescence

Paraffin-embedded pancreatic sections were stained with hematoxylin and eosin (H&E), and pathological changes were graded on a semi-quantitative scale from 0 (none) to 4 (severe) as previously described [12, 20, 21, 30]. Briefly, severity of pancreatic lesions was quantified as follows: 0, no pathological changes; 1, minimal infiltration of interstitial tissue with mononuclear cells; 2, moderate interstitial infiltration with mononuclear cells associated with beginning parenchymal destruction; 3, severe interstitial inflammation and/or progressive parenchymal destruction; 4, diffuse mononuclear cell infiltrates, extended destruction of pancreatic tissue, and replacement by adipose/fibrotic tissue. The samples were blinded prior to evaluation, and independently assessed by two experienced investigators. As a complementary method to verify infiltrates of T-cells, immunohistochemical detection of CD3^+^ cells in cryosections of pancreatic tissue was employed (data not shown).

Indirect immunofluorescence staining was performed using 6 μm thick sections of cryo-embedded pancreatic tissue from MRL/MpJ mice as described before [40]. Briefly, the tissue was fixed in ice-cold methanol for 10 min, washed with cold PBS for 2 × 10 min and blocked with 1 % bovine serum albumin (BSA; PAA Laboratories, Cölbe, Germany) in PBS-Tween 20 (0.1 %) for 30 min. Subsequently, the sections were incubated with the primary antibody (diluted 1:100 in 1 % BSA) for 1 h, washed with PBS and exposed to the fluorescence-labeled secondary antibody (diluted 1:600 in 1 % BSA) for an additional 60 min. After further washing and blocking steps, both incubations were repeated with different antibodies for double-staining. The following antibodies were used: primary antibodies: rat anti-mouse CD3 (BD Biosciences, Heidelberg, Germany), hamster anti-mouse CD11c (AbD Serotec, Puchheim, Germany); secondary antibodies: anti-rat AlexaFluor546 (red; Invitrogen, Karlsruhe, Germany), anti-hamster AlexaFluor488 (green; Invitrogen). Counterstaining was performed with 4′,6-Diamidino-2-phenylindole (DAPI; Sigma-Aldrich, Deisenhofen, Germany), and analyses were carried out on a Fluoview FV 10i microscope (Olympus, Tokyo, Japan) using the FV 10i Software (Olympus).

### Isolation of BM and cell culture of cDCs

MRL/MpJ and CAST/EiJ mice were used for generation of BM-derived cDCs following a standard protocol [31]. The animals were anesthetized with ketamine/xylazine, and the pancreas was harvested and cryo- and paraffin-embedded for further analyses. For the isolation of BM, femora and tibiae were removed and purified from surrounding muscle tissue. Afterwards, the condyles were cut off and the marrow was flushed with ice-cold PBS. The cell suspension was filtered through a 150 μm sieve and centrifuged at 300 g and 4°C for 10 min. After resuspension in cDC culture medium (RPMI-1640 [Biochrom, Berlin, Germany] supplemented with 10 % FCS [Biochrom], 1 % penicillin/streptomycin [Biochrom], 50 μM β-mercaptoethanol [Sigma-Aldrich] and 20 ng/ml GM-CSF [Immunotools, Friesoythe, Germany]), the cells were sowed at a density of 3 × 10^5^ cells/ml in 6 well-plates and grown at 37°C in a 5 % CO_2_ humidified atmosphere. On day 3 after BM isolation, the cells were diluted 1:2 by adding fresh cDC medium with GM-CSF to each well. On day 6 and 8, half of the medium was replenished with fresh GM-CSF-containing medium. To induce cDC maturation, the cells were harvested on day 9 after BM isolation by centrifugation, resuspended in cDC medium with GM-CSF and stimulated for 24 h with LPS at 1 μg/ml. Unstimulated cells were kept as controls. On day 10, the cells were harvested by centrifugation and subjected to flow cytometry analyses. The supernatants were collected and stored at −80°C for further analyses. The specific treatment protocols for individual assays are outlined below.

### Cytokine analyses

Cytokine concentrations in cDC culture supernatants were determined using the LEGENDplex Multi-Analyte Flow Assay Kit (BioLegend, San Diego, CA, USA), a bead-based immunoassay that quantifies multiple soluble analytes in biological samples simultaneously by flow cytometry. Specifically, the *Mouse Inflammation Panel* was employed to measure concentrations of CCL2 (MCP-1), GM-CSF, IFN-β, IFN-γ, IL-1α, IL-1β, IL-6, IL-10, IL-12 (p70), IL-17A, IL-23, IL-27 and TNF-α.

For the following proteins, concentrations below the detection limit of the assay were detected in most of the samples of all experimental groups: IFN-β, IFN-γ, IL-12, IL-17A, IL-23 and IL-27. In case of IL-12, measurements were subsequently repeated using the mouse IL-12 p70 DuoSet ELISA (R&D Systems, Minneapolis, MN, USA), which proved sensitive enough to quantify IL-12 levels in the culture supernatants. IFN-β, IFN-γ, IL-17A, IL-23 and IL-27 were not considered for further analyses, as it were GM-CSF (for the reason of exogenous supply) and IL-1β (the concentrations of which were a magnitude below the levels of IL-1α).

The assays were performed in 96-well plates, following the manufacturer's instructions. For measurements with the LEGENDplex Multi-Analyte Flow Assay Kit, a FACSVerse (BD Biosciences) was employed, and data were evaluated with the LEGENDplex™ Data Analysis software. IL-12 levels according to the DuoSet ELISA were recorded using a Glomax Multidetection System (Promega, Madison, WI, USA).

### Flow cytometry analyses of cDCs

On day 10, LPS-stimulated cDCs and unstimulated cells were harvested by centrifugation and resuspended in PBS. An average of 5 × 10^5^ cells per stain was subjected to subsequent analyses. Prior to staining, Fc receptors on cDCs were blocked by preincubation with anti-CD16/CD32 antibodies (Biolegend) for 5-10 min on ice. Surface staining was performed by incubating the cells with fluorochrome-conjugated specific antibodies, or the corresponding isotype controls, for at least 20 min in dark on ice. The following antibodies and isotype controls (unless stated otherwise, all purchased from Miltenyi Biotec, Bergisch Gladbach, Germany) were employed: anti-CD11c-FITC/-APC (#130-102-466/-493), anti-MHC-II-APC (#130-102-139), anti-CD40-PE (#130-102-599), anti-CD80-PE (#130-102-613), anti-CD83-PE (#130-104-474), anti-CD86-APC (#130-102-558), anti-hamster IgG-FITC/-PE/-APC (eBiosciences, San Diego, CA, USA), anti-rat IgG2a-PE (eBiosciences), anti-rat IgG2b-APC (Immunotools), and REA Control. Flow cytometric analyses were performed on a FACSCalibur (BD Biosciences). A total of 20,000 events per sample were acquired, and data were evaluated using the CellQuestPro software (BD Biosciences).

### Proliferation rate of cDCs

cDC proliferation was measured using a BrdU incorporation assay (Roche Applied Science, Mannheim, Germany). In short, cDCs from day 9 after BM isolation were sowed into 96-well plates at a density of 1.5 × 10^4^ cells/well and incubated with GM-CSF and/or LPS as indicated for 24 h. The next day, BrdU at 10 μM was added and incubation continued for another 24 h. Subsequently, BrdU incorporation was stopped and quantified *via* an enzyme-linked immunosorbent assay according to the manufacturer's instructions.

### Cocultures of cDCs and syngeneic T-cells

BM-derived cDCs from adult MRL/MpJ mice were harvested on day 9 after BM isolation and pretreated for 24 h with LPS (1 μg/ml) as indicated. Prior to coculture, the cells were irradiated with 30 Gy to block proliferation. T-cells were isolated from splenocytes of young and adult MRL/MpJ mice using magnetic cell separation (MACS) CD3 microbeads (Miltenyi Biotec) according to the manufacturer's instructions. As a result of optimization experiments, 1 × 10^5^ CD3^+^ T-cells and 5 × 10^3^ DCs per well were cocultured for 48 h in 96-well plates in cDC culture medium (± LPS as before). Afterwards, BrdU at 10 μM was added and incubation continued for another 24 h. BrdU incorporation was measured as described above and used as a surrogate marker of T-cell proliferation.

### Quantitative reverse transcriptase-PCR using real-time TaqMan^TM^ technology

cDCs, pretreated as indicated, were subjected to the isolation of RNA using TriFast reagent (PEQLAB Biotechnologie, Erlangen, Germany). Traces of genomic DNA were removed with the DNA-free kit. Afterwards, 250 ng of RNA was reverse transcribed into cDNA by means of TaqMan^TM^ Reverse Transcription Reagents and random priming (all reagents from Thermo Fisher Scientific, Darmstadt, Germany). Relative quantification of target cDNA levels by real-time PCR was performed in a ViiA 7 sequence detection system (Thermo Fisher Scientific). Therefore, qPCR MasterMix (Eurogentec, Seraing, Liège, Belgium) and mouse-specific TaqMan^TM^ Gene Expression Assays with fluorescently labeled MGB probes (Thermo Fisher Scientific) were used. The following assays were employed: Mm00454809_m1 (*cytip*), Mm04209424_g1 (*trem2*), Mm00488795_m1 (*clec4a2*), and Mm01545399_m1 (*hypoxanthine guanine phosphoribosyl transferase; hprt*; house-keeping gene control). PCR conditions were: 95°C for 10 min, followed by 40 cycles of 15 s at 95°C/1 min at 60°C. The relative amount of target mRNA was expressed as 2^−(Δ*Ct*)^, where ΔCt_target_ = Ct_target_ - Ct_hprt_.

### Statistical analyses

All data were analyzed using the IBM SPSS Statistics 22.0. Values were expressed as mean ± standard error of the mean (SEM) for the indicated number of separate cultures per experimental protocol. Statistical significance was checked using one-way ANOVA followed by Bonferroni's post-hoc test. If data did not meet the assumptions for ANOVA, analysis of variance was performed employing the Kruskal-Wallis test, before subgroups were tested using the Mann-Whitney U test. *P* < 0.05 (Bonferroni-adjusted as indicated in the figure legends) was considered to be statistically significant.

## SUPPLEMENTARY MATERIAL and FIGURES


