# Supplementary material for: In vitro studies implicate an imbalanced activation of dendritic cells in the pathogenesis of murine autoimmune pancreatitis
Source: Oncotarget. 2016 Jun 23;7(28):42963–77. doi: 10.18632/oncotarget.10265 (PMC5190000; doi:10.18632/oncotarget.10265)
Supplement: Supplementary file 1 [file oncotarget-07-42963-s001.pdf]

In vitro studies implicate an imbalanced activation of dendritic cells in the pathogenesis of murine autoimmune pancreatitis

Supplementary Material

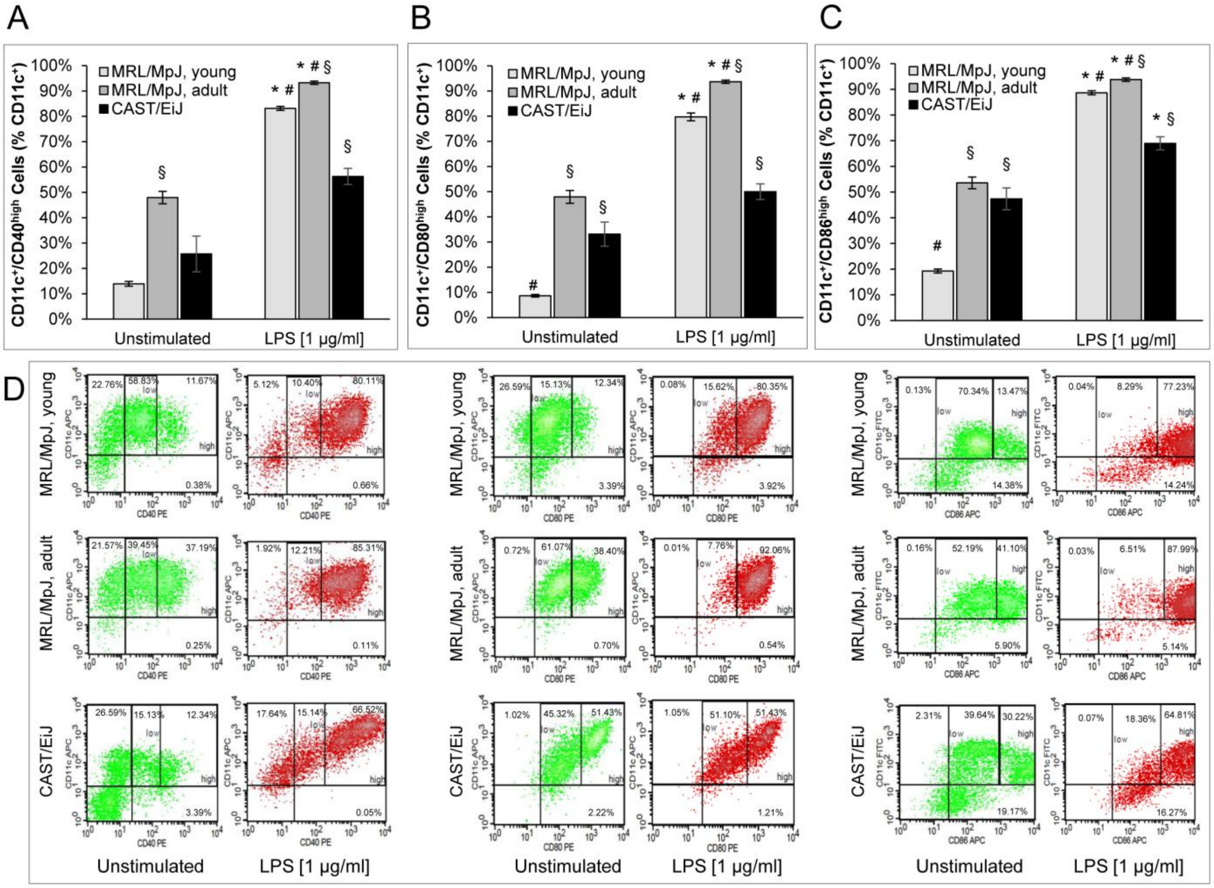

Fig. S1. Characterization of BM-derived cDCs by flow cytometry. cDCs of the indicated mouse cohorts were generated by culturing BM cells for 9 days with GM-CSF (20 ng/ml). Afterwards, the cells were stimulated for 24 h with LPS (1 µg/ml) as indicated. Expression of cell surface proteins was analyzed by flow cytometry. $CD11c^{+}/CD40^{high}$ (A), $CD11c^{+}/CD80^{high}$ (B) and $CD11c^{+}/CD86^{high}$ (C) cells are shown as percentage of all $CD11c^{+}$ cells (= 100 %). Data are presented as mean ± SEM (n ≥ 12 cultures from different mice); * $P < 0.05$ versus cDCs of the same experimental group cultured without LPS; § $P < 0.05$ versus identically cultured cDCs of young MRL/MpJ mice; # $P < 0.05$ versus identically cultured cDCs of CAST/EiJ mice. (D)

Representative dot plots of flow cytometric analyses are shown for all CD markers, experimental groups and culture conditions (with and without LPS, respectively).

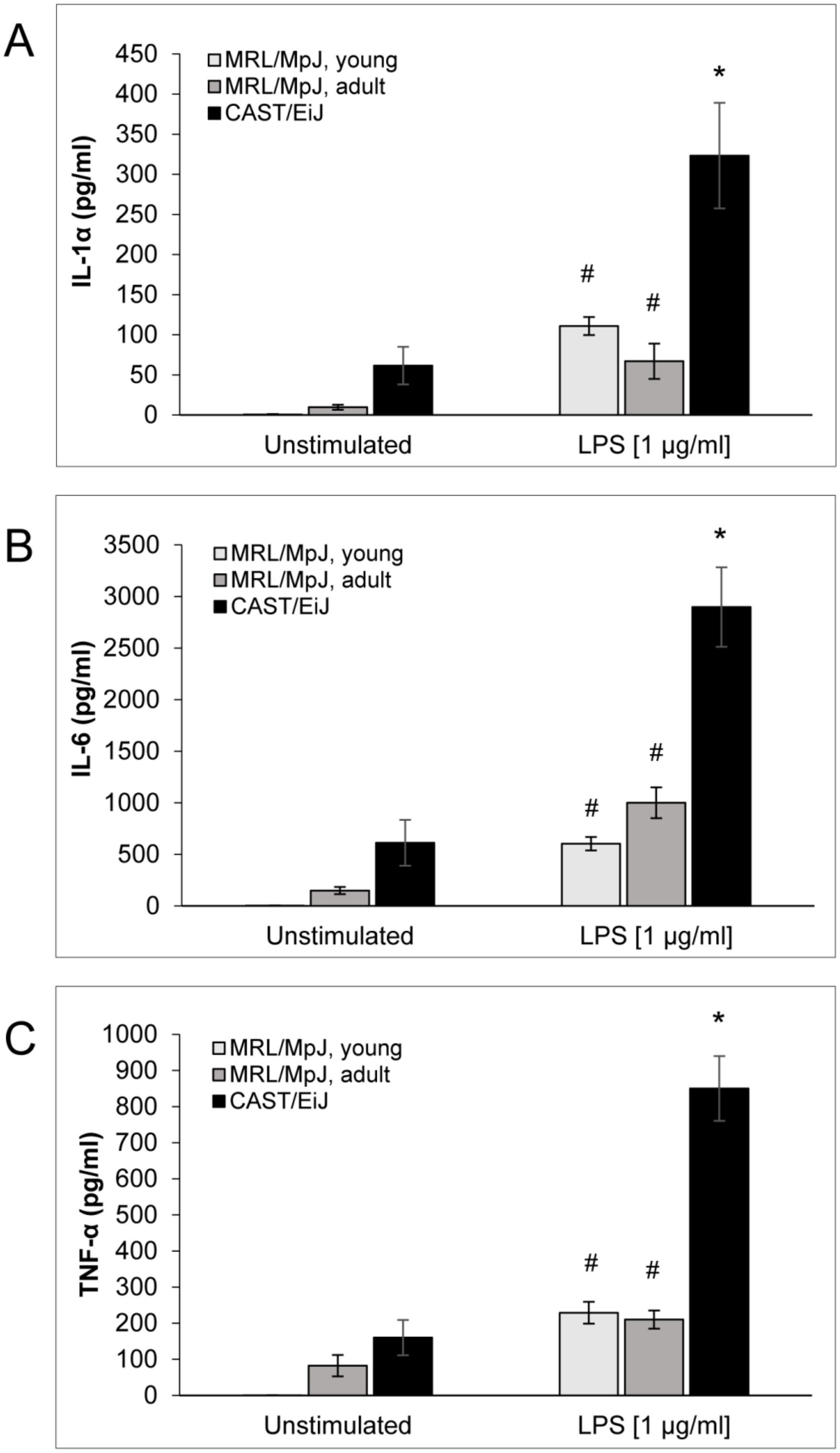

A
IL-1α (pg/ml)
450
400
350
300
250
200
150
100
50
0
MRL/MpJ, young
MRL/MpJ, adult
CAST/EiJ
*
#
#
Unstimulated
LPS [1 µg/ml]
B
IL-6 (pg/ml)
3500
3000
2500
2000
1500
1000
500
0
MRL/MpJ, young
MRL/MpJ, adult
CAST/EiJ
*
#
#
Unstimulated
LPS [1 µg/ml]
C
TNF-α (pg/ml)
1000
900
800
700
600
500
400
300
200
100
0
MRL/MpJ, young
MRL/MpJ, adult
CAST/EiJ
*
#
#
Unstimulated
LPS [1 µg/ml]

Fig. S2. Levels of IL-1α, IL-6 and TNF-α in cDC culture supernatants. After 9 days of routine culture with GM-CSF (20 ng/ml) alone, BM-derived cDCs of the indicated mouse cohorts were incubated with LPS for 24 h (1 µg/ml; right columns) or left unstimulated (left columns). Afterwards, the supernatants were subjected to the quantification of IL-1α (A), IL-6 (B) and TNF-α (C) levels using the LEGENDplex Multi-Analyte Flow Assay Kit. Data are presented as mean ± SEM (n = 6 cultures from different mice). * $P < 0.05$ versus identically cultured cDCs of young MRL/MpJ mice; $^{\#}P < 0.05$ versus identically cultured cDCs of CAST/EiJ mice. There were no significant differences between young and adult MRL/MpJ mice for any cytokine and experimental condition.
